# Organocatalytic asymmetric addition of malonates to unsaturated 1,4-diketones

**DOI:** 10.3762/bjoc.8.165

**Published:** 2012-09-04

**Authors:** Sergei Žari, Tiiu Kailas, Marina Kudrjashova, Mario Öeren, Ivar Järving, Toomas Tamm, Margus Lopp, Tõnis Kanger

**Affiliations:** 1 Department of Chemistry, Tallinn University of Technology, Akadeemia tee 15, 12618 Tallinn, Estonia

**Keywords:** Michael addition, non-covalent catalysis, organocatalysis, squaramide, thiourea

## Abstract

The organocatalytic Michael addition of malonates to symmetric unsaturated 1,4-diketones catalyzed by thiourea and squaramide derivatives with *Cinchona* alkaloids afforded the formation of a new C–C bond in high yields (up to 98%) and enantiomeric purities (up to 93%). The absolute configuration of the product was suggested from comparison of the experimental and calculated VCD spectra of the reaction product **3a**.

## Introduction

The asymmetric 1,4-conjugated addition (Michael reaction) of C-nucleophiles to enones is a powerful tool for obtaining a significant variety of enantioenriched products through a carbon–carbon bond formation [1–5]. Recently, unsaturated 1,4-dicarbonyl compounds, such as 1,4-ketoesters [6–8], 1,4-diketones [9], 1,4-ketoamides [9–10] and dialkylfumarates [11], have been the substrates for this reaction. The reaction products can undergo further chemical transformations, allowing the possibility of cascade reactions, making the method attractive for the synthesis of several valuable compounds, such as drugs and natural products. Tan et al. performed the addition of 1,3-alkylthiomalonates to 1,4-dicarbonylbut-2-enes, catalyzed by chiral bicyclic guanidines [7,9,12]. Xiao et al. reported the addition of nitroalkanes to 4-oxo-enoates, using chiral urea derivatives [7]. Miura et al. achieved an asymmetric addition of α,α-disubstituted aldehydes to maleimides catalyzed by primary amine thiourea organocatalyst [13]. Wang et al. reported the addition of dialkylmalonates and nitromethane to 4-oxo-4-arylbutenoates catalyzed by *N,N´*-dioxide-Sc(OTf)_3_ complexes [8]. Despite these and other successful experimental results, the asymmetric addition of malonates to symmetric aromatic unsaturated 1,4-diketones has not been systematically studied. Products of that reaction can be used as precursors of biologically active compounds. Padmaja et al. have reported that racemic heterocyclic compounds derived from the Michael addition of malonates and malononitrile to unsaturated 1,4-diketones possess antimicrobial and antifungal properties [14–15]. Therefore, new asymmetric additions of C-nucleophiles to unsaturated 1,4-diketones are highly in demand.

**Figure 1:**
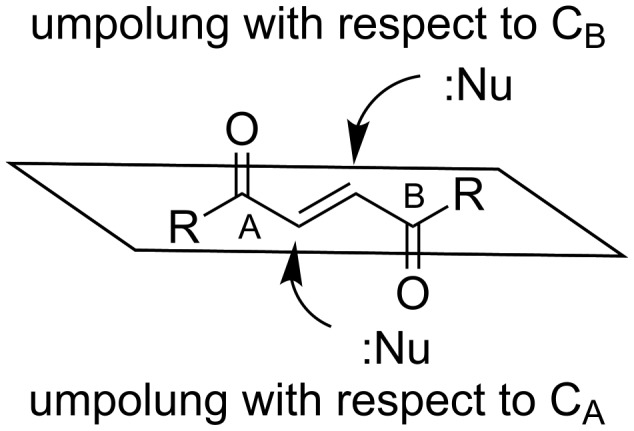
The conjugated addition to unsaturated 1,4-diketone **1**.

The asymmetric desymmetrization of symmetric unsaturated 1,4-diketones is a very challenging target. *si*-Attack on one carbon atom of the double bond and *re*-attack on the other leads to the same enantiomer. From the synthetic point of view, the conjugate addition of the nucleophile is, at the same time, a formal umpolung reaction with respect to the other carbonyl group (Figure 1).

## Results and Discussion

### Catalyst screening

**Figure 2:**
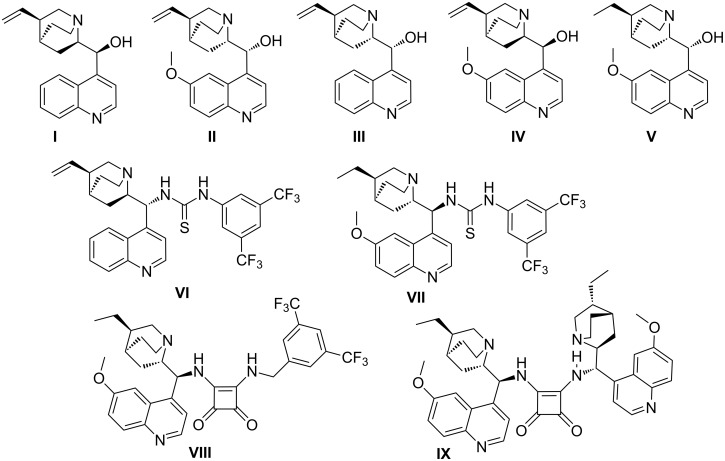
Organocatalysts screened.

As a part of our ongoing studies in organocatalysis [16–19] we investigated the organocatalytic approach to the asymmetric desymmetrization of the title compounds with malonates. Three types of organocatalysts providing noncovalent interactions were used for this purpose: *Cinchona* alkaloids (**I**–**V**), thiourea derivatives (**VI**, **VII**) and squaramide derivatives (**VIII**, **IX**) (Figure 2). All of these screened catalysts are bifunctional compounds possessing hydrogen-bonding donor and acceptor moieties. Catalysts based on thiourea and squaramide differ from each other in their possible hydrogen-bond angles, rigidity of conformation, and p*K*_a_ values [20]. Although the two squaramide based catalysts **VIII** and **IX** are structurally similar, they have quite different properties. Catalyst **IX** is a self-association-free compound [21], while catalyst **VIII** forms associates, and the stereoselectivity of the reaction in its presence depends on the catalyst concentration [22].

The catalysts were screened in the reaction of phenyl disubstituted unsaturated 1,4-diketone **1a** with diethyl malonate (**2a**, Table 1). The reaction was run in DCE at room temperature in the presence of 10 mol % of catalyst with a five-fold excess of malonate. In all cases, the yields of the products were very high. *Cinchona* alkaloids (Table 1, entries 1–4) catalyzed the reaction with low stereoselectivity. There was a remarkable difference in their reaction rates. Quinine (**II**) and quinidine (**IV**, Table 1, entries 2 and 4) were more efficient than cinchonine (**I**) and cinchonidine (**III**, Table 1, entries 1 and 3). The reduction of the vinyl group in quinine afforded dihydroquinine **V**. Unfortunately, no changes in the stereoselectivity of the model reaction were observed (Table 1, entry 5). Both thiourea catalysts derived from *Cinchona* alkaloids (**VI**, **VII**) gave high yields with good selectivities (Table 1, entries 6 and 7). Squaramide **VIII** and *C*_2_-symmetric squaramide **IX** gave good yields but slightly lower selectivities (Table 1, entries 8 and 9). The catalyst **VII** was selected for further studies as being the most efficient. Also, considering the partially aromatic character of the cyclobutenedione system, which may possibly allow additional interactions with the aromatic substrates **1**, the catalyst **IX** was also chosen.

**Table 1:** Screening of the catalysts for the asymmetric conjugated-addition reaction.

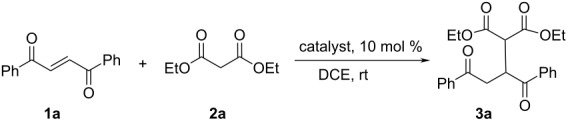					

entry	catalyst	time (h)	yield^a^ (%)	ee^b^ (%)	abs. conf.^c^

1	**I**	120	97	13	R
2	**II**	28	98	27	R
3	**III**	168	88	10	R
4	**IV**	24	>98	19	S
5	**V**	20	>98	18	R
6	**VI**	24	>98	74	S
7	**VII**	19	98	74	R
8	**VIII**	15	96	60	R
9	**IX**	96	96	39	R

^a^Isolated yield; ^b^determined by chiral HPLC; ^c^determined by a comparison of the experimental and calculated VCD spectra (see the following).

### Scope of the reaction

Next, we studied the effect of the malonate structure on the stereoselectivity of the reaction (Table 2). Although, the ester moiety can be replaced by other functional groups in the course of further synthetic transformations its main role is to provide the addition products with high ee value. The conditions for the reaction remained the same as they were in the catalyst screening experiments, except that a smaller excess of malonate (3 equiv, unless stated otherwise) was used. This did not influence the reaction time or the enantioselectivity, but afforded easier purification of the crude product.

**Table 2:** Enantioselective addition of malonates **2a**-**2f** to unsaturated 1,4-diketone **1a**.

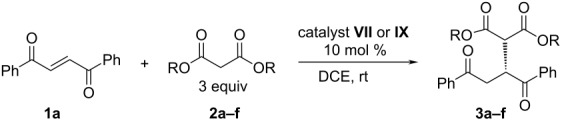					

entry	R	catalyst	time (h)	yield^c^ (%)	ee^d^ (%)

1	**a:** Et	**VII**	19	99	74
2	**a:** Et	**IX**	96	96	39
3	**b:** Me	**VII**	15	92	73
4	**b:** Me	**IX**	30	95	87
5	**c:** iPr	**VII**	36	96	69
6	**c:** iPr	**IX**	96	96	60
7^a^	**d:** *t*-Bu	**VII**	75	70	59
8^a^	**d:** *t*-Bu	**IX**	33	95	70
9^b^	**e:** Ph	**VII**	3	44	37
10^b^	**e:** Ph	**IX**	2	41	81
11	**f:** Bn	**VII**	6	95	68
12	**f:** Bn	**IX**	6	92	84

^a^Reaction at 80 °C, malonate/dione 2:1; ^b^malonate/dione 1:1; ^c^yields of isolated products; ^d^determined by chiral HPLC.

1,4-Diketone **1a** reacted smoothly with a variety of malonates **2a**–**2f**, affording the products **3a**–**3f** in high yields and with moderate to high stereoselectivities. In the case of the catalyst **VII**, the increase of steric hindrance of the malonate (Table 2, entries 1, 3, 5 and 7) led to a gradual drop in selectivity. Sterically more demanding malonates with branched alkyl or aryl groups (**2c–e**) gave products in much lower enantioselectivity (ee 37–69%) than the simple alkyl malonates (**2a, b)** (ee 73–74%). There was no clearly observed similar dependence with squaramide catalyst **IX**. Almost equally high ee values were obtained with methyl, phenyl or benzyl malonates (Table 2, entries 4, 10 and 12). A possible reason for the high selectivity with the phenyl-ring-containing esters could be the aromatic nature of the squaramide functional group in catalyst **IX**, allowing additional π–π-interactions.

The properties of the enone double bond of the substrate depend on the nature of the substituents in the phenyl ring. Therefore, the electronic effect of the *para***-**substituent of unsaturated 1,4-diketone **1** on the reaction was investigated (Table 3). Electron-withdrawing groups, such as bromo and nitro, (Table 3, entries 10 and 13) as well as the electron-donating methoxy group (Table 3, entry 7) led to an increase in stereoselectivity, but the reaction time was also increased and the yields were lower with catalyst **VII**. A methyl substituent slightly decreased the enantioselectivity (Table 3, entry 4). This means that these dependencies cannot be clearly rationalized by the use of the electronic effects of the substituents in the phenyl ring. In the case of squaramide catalyst **IX**, all of the reactions became sluggish at room temperature (Table 3, entries 5, 8, 11 and 14). The reaction times were unreasonably long and the yields remained low. However, in the case of reactions with electron-withdrawing groups, the enantiomeric purity of the products was higher (Table 3, entries 11 and 14).

**Table 3:** Enantioselective addition of diethylmalonate **2a** to substituted 1,4-diketones **1a, g–j**.

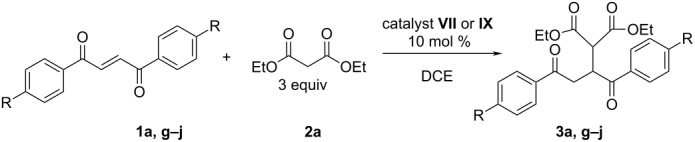						

entry	R	catalyst	temp. (°C)	time (h)	yield^b^ (%)	ee^c^ (%)

1	**a**: H	**VII**	rt	19	99	74
2	**a**: H	**IX**	rt	96	97	39
3	**a**: H	**IX**	80	10	97	82
4	**g**: Me	**VII**	rt	18	96	69
5	**g**: Me	**IX**	rt	213	25	18
6	**g**: Me	**IX**	80	22	76	66
7	**h**: MeO	**VII**	rt	88	90	79
8	**h**: MeO	**IX**	rt	48	—	—
9	**h**: MeO	**IX**	80	22	90	83
10^a^	**i**: Br	**VII**	rt	48	83	81
11^a^	**i**: Br	**IX**	rt	123	20	93
12^a^	**i**: Br	**IX**	80	6	86	91
13	**j**: NO_2_	**VII**	rt	54	66	81
14	**j**: NO_2_	**IX**	rt	94	44	89
15	**j**: NO_2_	**IX**	80	6	98	89

^a^Dione/malonate 1.2:1; ^b^yields of isolated products; ^c^determined by chiral HPLC.

Increasing the temperature had a drastic positive effect on the reactions performed in the presence of catalyst **IX**. It was found that by raising the temperature to 80 °C it was possible to significantly decrease the reaction time and increase yields up to 98% with almost no negative effect on the stereoselectivity (ee 66–89%, Table 3, entries 6, 9, 12 and 15). Moreover, the compounds **3a** and **3g** were obtained in much higher enantioselectivities, and the reaction with the unsaturated 1,4-diketone containing the electron-donating substituent **1h**, which did not react at room temperature, also afforded the product in good yield and selectivity (Table 3, entry 9). As the squaramide-type catalyst **IX** is known to be self-association-free [21], the increase in enantioselectivity at higher temperatures can be attributed to the thermodynamic control of the conjugate addition. At the same time, the increase in temperature resulted in a small drop in stereoselectivity for the model reaction with catalyst **VII**.

**Figure 3:**
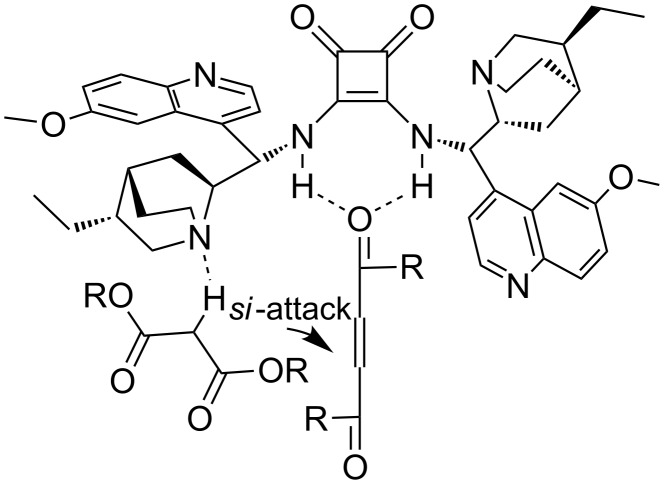
Proposed transition state.

The mechanism of the reaction is believed to be similar to that previously reported for 1,3-dicarbonyl compounds and acyl phosphonates [23]. Squaramide **IX** is a bifunctional catalyst that simultaneously coordinates electrophilic unsaturated 1,4-diketone via hydrogen bonding and activates the nucleophilic malonate via the tertiary amine of the quinuclidine moiety. Due to the symmetry of the substrate, there is no regioselectivity problem. A face selection is determined by the different access of the nucleophile to the tertiary amino group between the side chains of the catalysts. The *re*-face of the Michael acceptor is shielded by the flat quinoline unit and the *si*-attack of the malonate is preferred, affording *R*-selectivity (Figure 3).

### Determination of the absolute configuration

**Figure 4:**
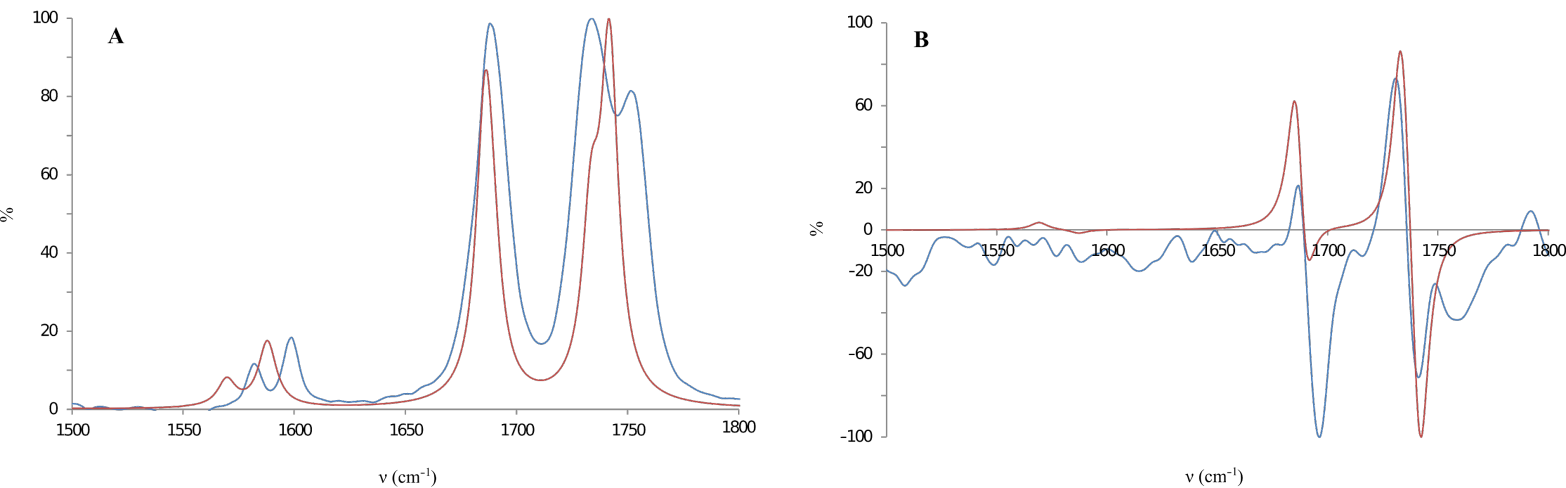
Calculated (red) and experimental (blue) IR (A) and VCD spectrum (B) of compound (*R*)-**3a**.

The absolute configuration of the product **3a** was determined by a comparison of the experimental and calculated vibrational circular dichroism (VCD) spectra. DFT calculations (method B3PW91/6-311G**) of a series of conformers of compound **3a** with *R*-configuration were performed. Calculations of harmonic vibrational frequencies were carried out for all favored conformers to verify their stability. The Boltzmann distribution of the Gibbs energy showed that one conformation out of six is dominant (84%). The experimental and calculated IR spectra match well in the range 1500–1800 cm^−1^ (both experimental and calculated spectra are normalized to 100% by using the highest peak from that range, Figure 4A).

The most characteristic peaks of VCD spectra are in the same region (Figure 4B). The good agreement between calculated and experimental spectra directly allows for the assignment of the absolute configuration of **3a** as the *R*-enantiomer.

## Conclusion

We have developed a highly enantioselective method for the desymmetrization of aromatic unsaturated 1,4-diketones through organocatalytic reactions with malonates. The reaction is catalyzed by thiourea and squaramide derivatives with *Cinchona* alkaloids and affords products in very high yields (up to 99%) and in high enantioselectivities (up to 93%). This enantioselective 1,4-addition to unsaturated 1,4-diketones affords valuable intermediates for further synthetic transformations.

## Supporting Information

File 1 Experimental procedures, compound characterization and computational data.
