# Supplementary material for: Organocatalytic asymmetric addition of malonates to unsaturated 1,4-diketones
Source: Beilstein J Org Chem. 2012 Sep 4;8:1452–7. doi: 10.3762/bjoc.8.165 (PMC3458770; doi:10.3762/bjoc.8.165)
Supplement: File 1 — Experimental procedures, compound characterization and computational data. [file Beilstein_J_Org_Chem-08-1452-s001.pdf]

Supporting Information
for

Organocatalytic asymmetric addition of malonates to unsaturated 1,4-diketones

Sergei Žari, Tiiu Kailas, Marina Kudrjashova, Mario Öeren, Ivar Järving, Toomas Tamm, Margus Lopp and Tõnis Kanger*

Address: Department of Chemistry, Tallinn University of Technology, Akadeemia tee 15, 12618 Tallinn, Estonia

Email: Tõnis Kanger* - kanger@chemnet.ee

* Corresponding author

Experimental procedures, compound characterization and computational data

General

Full assignment of ^{1}H and ^{13}C chemical shifts is based on the 1D and 2D FT NMR spectra on a Bruker Avance^{III} 400 instrument. Internal standard (TMS $\delta = 0.00$) and solvent peak ($CHCl_3$ $\delta = 77.16$) were used as chemical shift references. HRMS spectra were recorded by using Agilent Technologies 6540 UHD Accurate-Mass Q-TOF LC/MS spectrometer by using AJ-ESI ionization. Chiral HPLC was performed by using Chiralpak AS-H (250 × 4.6 mm), Chiralpak AD-H (250 × 4.6 mm), Chiralcel OJ-H (250 × 4.6 mm) or Lux 3u Amylose-2 (250 × 4.6 mm) columns. Optical rotations were obtained by using an Anton Paar GWB Polarimeter MCP500. IR and VCD spectra were recorded on a Bruker PMA 50 accessory coupled to a Tensor 27 Fourier transform infrared spectrometer. A transmission cell equipped with BaF_2 windows of 0.1 mm optical path length was used. The VCD probe was prepared by dissolving the (−)-enantiomer of **3a** (9.5 mg; ee = 74%) in CCl_4 (300 µL) yielding a 2% solution (80 mM). The baseline of the spectrum was corrected (by subtracting the VCD spectrum of CCl_4, measured under the same experimental conditions, from the VCD spectrum of sample). For the VCD spectrum 21300 scans were averaged at 4 cm^{-1} resolution

(corresponding to 5 h measurement time). The spectra are presented without smoothing and further data processing. MS spectra were measured on a Shimadzu GSMS-QP2010 spectrometer at 70 eV EI. Precoated silica gel 60 F_{254} plates were used for TLC, whereas for column chromatography silica gel KSK40–100 µm was used. Commercial reagents were generally used as received.

Synthesis of starting materials

Synthesis of unsaturated 1,4-diketones

All 1,4-diketones except for **1j** were prepared by a method proposed by Conant and Lutz [1] and their spectral data match with those in the literature.

***trans*-1,4-Di(*p*-nitrophenyl)-2-butene-1,4-dione (1j)** was prepared by a two-step procedure. In the first step, *p*-nitroacetophenone was transformed to *p*-nitro-α-oxo-benzeneacetaldehyde by a procedure described in literature [2]. In the second step, *p*-nitro-α-oxo-benzeneacetaldehyde (375 mg; 2.09 mmol) was dissolved in dry DCM (10 mL). A DCM solution of 1-*p*-nitrophenyl-2-triphenylphosphoranylidene-ethanone (1.2 g; 2.8 mmol/10 mL) was added dropwise. After 10 min a solid started to precipitate. The reaction was followed by TLC. After completion of the reaction, the mixture was filtered, and the solid was washed with cold chloroform. The solid was recrystallized from the mixture of chloroform and ethyl acetate affording the product (450 mg, 66% yield). The spectral data of the product matched those in the literature.

Synthesis of malonates

Malonates (**2a**–**c**, **2f**) [3], **2d** [4], and **2e** [5] were prepared by corresponding literature procedures and their spectral data matched those of the literature.

Synthesis of catalysts

Catalysts **I–IV** were commercially available from Aldrich and were used as received. Catalysts **V** [6], **VI, VII** [7], **VIII** [8] and **IX** [9] were synthesized according to literature procedures and spectral data matched those of the literature.

General Procedure for the asymmetric conjugate addition of malonates 2 to unsaturated diketones 1

A mixture of unsaturated 1,4-diketone **1** (0.25 mmol), malonate **2** (0.75 mmol) and catalyst (0.025 mmol) in DCE (100 µL) was stirred at room temperature until TLC showed that 1,4-diketone reacted away. The product was purified by column chromatography using heptane-EtOAc as eluent.

(*R*)-Diethyl 2-(1,2-dibenzoylethyl)propanedioate (3a)

A crude product was purified by column chromatography on silica gel (heptane/EtOAc 8:1) affording colorless oil that became solid upon standing in the fridge. Mp= 68-69 °C. Enantiomeric excess was determined by HPLC (Chiralpack AS-H column, 9:1 hexane/iPrOH, 1 mL/min, 25 °C, 254 nm). Major isomer t_r = 17.90 min, minor isomer t_r = 13.72 min). $[\alpha]_D^{25}$ = −17.9° (ee = 74%; *c* 0.22; MeOH); IR (KBr) *v*: 3062, 2983, 1731, 1682, 1598, 1449, 1369, 1226, 1183, 1159, 1034, 753, 690. MS *m*/*z*: 396 (M), 291, 245, 105, 77; HRMS: calcd for $C_{23}H_{24}O_6$ $[M + H]^+$ 397.1646, found 397.1630; ^1H NMR (400 MHz, $CDCl_3$) δ 8.06–8.03 (m, 2H), 7.93–7.89 (m, 2H), 7.58–7.55 (m, 2H), 7.49–7.41 (m, 4H), 4.92 (ddd, *J* = 8.70, 6.14, 5.95 Hz, 1H), 4.19–4.00 (m, 4H), 3.92 (d, *J* = 8.72 Hz, 1H), 3.59 (dd, *J* = 18.20, 5.88 Hz, 1H), 3.41 (dd, *J* = 18.18, 6.22 Hz, 1H), 1.19 (t, *J* = 7.14 Hz, 3H), 1.15 (t, *J* = 7.14 Hz, 3H); ^{13}C NMR (101 MHz, $CDCl_3$) δ 200.29, 196.58, 168.31, 168.02, 136.15, 135.72, 133.43, 133.33, 128.77, 128.72, 128.63, 128.12, 61.83, 61.76, 53.16, 40.51, 38.97, 13.87, 13.82.

Dimethyl 2-(1,2-dibenzoylethyl)propanedioate (3b)

A crude product was purified by column chromatography on silica gel (heptane/EtOAc 5:1) affording colorless oil. Enantiomeric excess was determined by HPLC (Chiralpack AS-H column, 9:1 hexane/iPrOH, 1 mL/min, 25 °C, 254 nm). Major isomer t_r = 26.74 min, minor isomer t_r = 16.68 min). $[\alpha]_D^{25}$ = −20.8° (ee = 87%; *c* 0.25; $CHCl_3$); IR (KBr) *v*: 2954, 1733, 1682, 1597, 1449, 1226, 1025, 753, 691; MS *m*/*z*: 368 (M^+), 319, 305, 263, 231, 199, 105, 77; HRMS: calcd for $C_{21}H_{20}O_6$ $[M + H]^+$ 369.1333, found 369.1320; ^1H NMR (400 MHz, $CDCl_3$) δ 8.07–8.01 (m, 2H), 7.94–7.88 (m, 2H), 7.60–7.53 (m, 2H), 7.51–7.40 (m, 4H), 4.92 (dt, *J* = 8.7, 6.1 Hz, 1H), 3.95 (d, *J* = 8.7 Hz, 1H), 3.66 (s, 1H), 3.62 (s, 1H), 3.60 (dd, *J* = 18.2, 6.2 Hz, 1H), 3.39 (dd, *J* = 18.2, 5.9 Hz, 1H); ^{13}C NMR (101 MHz, $CDCl_3$) δ 200.18, 196.54, 168.69, 168.46, 136.08, 135.51, 133.48, 133.42, 128.76, 128.74, 128.64, 128.12, 52.77, 52.74, 52.71, 40.70, 38.95.

Diisopropyl 2-(1,2-dibenzoylethyl)propanedioate (3c)

A crude product was purified by column chromatography on silica gel (heptane/EtOAc 10:1) affording colorless oil that became solid at 5 °C after the addition of a small amount of MeOH. Mp 69–70 °C. Enantiomeric excess was determined by HPLC (Chiralpack AS-H column, 9:1 hexane/iPrOH, 1 mL/min, 25 °C, 254 nm). Major isomer t_r = 13.98 min, minor isomer t_r = 11.52 min); $[\alpha]_D^{25}$ = -13.8° (ee = 69%; *c* 0.25; $CHCl_3$); IR (KBr) *v*: 2982, 1725, 1685, 1450, 1374, 1252, 1183, 1104, 691; MS *m*/*z*: 424 (M), 347, 319, 305, 259, 218, 173,

105, 77; HRMS: calcd for $C_{25}H_{28}O_6$ [M + H]$^+$ 425.1959, found 425.1943; ^{1}H NMR (400 MHz, $CDCl_3$) δ 8.07–8.04 (m, 2H), 7.92–7.88 (m, 2H), 7.58–7.52 (m, 2H), 7.49–7.40 (m, 4H), 4.98 (sep, *J* = 6.30Hz, 1H), 4.93 (sep, *J* = 6.33 Hz, 1H), 4.89 (ddd, *J* = 8.74, 6.50, 5.61 Hz, 1H), 3.86 (d, *J* = 8.70 Hz, 1H), 3.57 (dd, *J* = 18.16, 5.48 Hz, 1H), 3.41 (dd, *J* = 18.14, 6.58 Hz), 1H), 1.24 (d, *J* = 6.28 Hz, 3H), 1.20 (d, *J* = 6.28 Hz, 3H), 1.16 (d, *J* = 6.28 Hz, 3H), 1.12 (d, *J* = 6.28 Hz, 3H); ^{13}C NMR (101 MHz, $CDCl_3$) δ 200.40, 196.66, 167.84, 167.54, 136.25, 135.91, 133.36, 133.25, 128.81, 128.68, 128.60, 128.11, 69.52, 69.44, 53.62, 40.30, 38.95, 21.56, 21.53, 21.51, 21.33.

Di-*tert*-butyl 2-(1,2-dibenzoylethyl)propanedioate (3d)

A crude product was purified by column chromatography on silica gel (heptane/EtOAc 15:1) affording colorless oil. A white solid precipitated upon storage at 5 °C. Mp 68–69 °C. Enantiomeric excess was determined by HPLC (Chiralpack AS-H column, 98:2 hexane/iPrOH, 1 mL/min, 25 °C, 254 nm). Major isomer t_r = 19.07 min, minor isomer t_r = 7.99 min). $[\alpha]_D^{25}$ = −4.5° (ee = 70%; *c* 0.25; $CHCl_3$); IR (KBr) *v*: 2979, 1725, 1685, 1450, 1255, 1143, 1004, 848, 752, 691; MS *m*/*z*: 429, 334, 296, 279, 248, 191, 174, 146, 105, 77; HRMS: calcd for $C_{27}H_{32}O_6$ [M + Na]$^+$ 475.2091, found 475.2087; ^{1}H NMR (400 MHz, $CDCl_3$) δ 8.07–8.04 (m, 2H), 7.92–7.89 (m, 2H), 7.57–7.51 (m, 2H), 7.49–7.40 (m, 4H), 4.83 (ddd, *J* = 8.52, 6.99, 5.04 Hz, 1H), 3.73 (d, *J* = 8.56 Hz, 1H), 3.55 (dd, *J* = 18.12, 5.00 Hz, 1H), 3.43 (dd, *J* = 18.12, 7.00 Hz, 1H), 1.42 (s, 9H), 1.34 (s, 9H); ^{13}C NMR (101 MHz, $CDCl_3$) δ 200.49, 196.83, 167.57, 167.13, 136.33, 136.09, 133.27, 133.13, 128.83, 128.65, 128.57, 128.13, 82.30, 81.12, 55.14, 40.29, 38.83, 27.80, 27.72.

Diphenyl 2-(1,2-dibenzoylethyl)propanedioate (3e)

A crude product was purified by column chromatography on silica gel (heptane/EtOAc 10:1) affording a yellowish solid (mp 72–75 °C). Enantiomeric excess was determined by HPLC (Chiralpack AS-H column, 9:1 hexane/iPrOH, 1 mL/min, 25 °C, 254 nm). Major isomer t_r = 24.06 min, minor isomer t_r = 21.55 min. $[\alpha]_D^{25}$ = −84.0° (ee= 81%; *c* 0.26; $CHCl_3$); IR (KBr) *v*: 3062, 2925, 1751, 1681, 1491, 1449, 1185, 1002, 747, 687, 496; MS *m*/*z*: 399, 354, 305, 278, 260, 233, 105, 94, 77, 66; HRMS: calcd for $C_{31}H_{24}O_6$ [M + H]$^+$ 493.1646, found 493.1643; ^{1}H NMR (400 MHz, $CDCl_3$) δ 8.11–8.07 (m, 2H), 7.95–7.91 (m, 2H), 7.61–7.52 (m, 2H), 7.51–7.40 (m, 4H), 7.40–7.33 (m, 4H), 7.25–7.20 (m, 2H), 7.18–7.13 (m, 2H), 7.12–7.08 (m, 2H), 5.18 (ddd, *J* = 7.72, 7.03, 5.12 Hz, 1H), 4.42 (d, *J* = 7.76 Hz, 1H), 3.86 (dd, *J* = 18.48, 6.92 Hz), 3.48 (dd, *J* = 18.48, 5.08 Hz, 1H); ^{13}C NMR (101 MHz, $CDCl_3$) δ 199.88, 196.54, 166.92, 166.78, 150.42, 150.35, 136.05, 135.27, 133.61, 129.54, 129.51, 128.91, 128.85, 128.69, 128.19, 126.36, 126.28, 121.34, 121.28, 52.57, 40.82, 38.62.

Dibenzyl 2-(1,2-dibenzoylethyl)propanedioate (3f)

A crude product was purified by column chromatography on silica gel (heptane/EtOAc 10:1) affording colorless oil that turned solid after the addition of small amount of MeOH (mp 66–67 °C). Enantiomeric excess was determined by HPLC (Lux 3u Amylose-2 column, 6:4 hexane/iPrOH, 1 mL/min, 35 °C, 254 nm). Major isomer t_r = 57.48 min, minor isomer t_r = 50.06 min. $[\alpha]_D^{25}$ = −23.4° (ee = 84%; *c* 0.25; $CHCl_3$); IR (KBr) *v*: 3064, 3034, 2955, 1733, 1683, 1597, 1450, 1261, 1226, 1158, 1003, 751, 697; MS *m/z*: 368, 233, 215, 205, 190, 127, 105, 91, 77; HRMS: calcd for $C_{33}H_{28}O_6$ $[M + H]^+$ 521.1959, found 521.1933; ^1H NMR (400 MHz, $CDCl_3$) δ 8.03–7.99 (m, 2H), 7.84–7.80 (m, 2H), 7.57–7.51 (m, 2H), 7.47–7.37 (m, 4H), 7.29–7.24 (m, 6H), 7.24–7.20 (m, 2H), 7.20–7.17 (m, 2H), 5.11 (d, *J* = 12.20 Hz, 1H), 5.04 (d, *J* = 12.28 Hz, 1H), 5.01 (d, *J* = 12.20 Hz, 1H), 5.00 (d, *J* = 12.28 Hz, 1H), 4.94 (ddd, *J* = 8.35, 6.19, 5.96 Hz, 1H), 4.04 (d, *J* = 8.36 Hz, 1H), 3.54 (dd, *J* = 18.28, 5.88 Hz), 3.38 (dd, *J* = 18.28, 6.28 Hz, 1H); ^{13}C NMR (101 MHz, $CDCl_3$) δ 200.08, 196.54, 167.98, 167.77, 136.06, 135.58, 134.97, 134.95, 133.41, 133.36, 128.77, 128.73, 128.59, 128.51, 128.47, 128.38, 128.30, 128.18, 128.14, 67.58, 67.49, 53.01, 40.66, 38.83.

Diethyl 2-(1,2-ditoluoylethyl)propanedioate (3g)

A crude product was purified by column chromatography on silica gel (heptane/EtOAc 8:1) affording colorless oil that was recrystallized from acetone/hexane (mp 74–75 °C). Enantiomeric excess was determined by HPLC (Chiralpack AS-H column, 9:1 hexane/iPrOH, 1 mL/min, 25 °C, 254 nm). Major isomer t_r = 13.83 min, minor isomer t_r = 11.09 min. $[\alpha]_D^{25}$ = −9.9° (ee = 69%; *c* 0.25; $CHCl_3$); IR (KBr) *v*: 2983, 1731, 1680, 1607, 1409, 1369, 1235, 1181, 1035, 816; MS *m/z*: 424 (M), 361, 333, 305, 259, 119, 91, 65; HRMS: calcd for $C_{25}H_{28}O_6$ $[M + H]^+$ 425.1959, found 425.1970; ^1H NMR (400 MHz, $CDCl_3$) δ 7.96–7.93 (m, 2H), 7.82–7.78 (m, 2H), 7.27–7.24 (m, 2H), 7.24–7.20 (m, 2H), 4.89 (ddd, *J* = 8.86, 6.06, 6.05 Hz, 1H), 4.18–4.00 (m, 4H), 3.90 (d, *J* = 8.84 Hz, 1H), 3.55 (dd, *J* = 18.14, 6.06 Hz, 1H), 3.34 (dd, *J* = 18.12, 6.00 Hz, 1H), 2.40 (s, 3H), 2.39 (s, 3H), 1.18 (t, *J* = 7.13 Hz, 3H), 1.15 (t, *J* = 7.11 Hz, 3H); ^{13}C NMR (101 MHz, $CDCl_3$) δ 199.98, 196.16, 168.44, 168.11, 144.24, 144.18, 133.76, 133.16, 129.41, 129.29, 128.92, 128.24, 61.77, 61.70, 53.29, 40.37, 38.98, 21.68, 21.66, 13.87, 13.84.

Diethyl 2-(1,2-di(*p*-methoxybenzoyl)ethyl)propanedioate (3h)

A crude product was purified by column chromatography on silica gel (heptane/EtOAc 10:1) affording a yellowish oil. Enantiomeric excess was determined by HPLC (Chiralpack AS-H column, 8:2 hexane/iPrOH, 1 mL/min, 25 °C, 254 nm). Major isomer t_r = 23.36 min, minor

isomer t_r = 20.79 min. $[\alpha]_D^{25}$ = +6.7° (ee = 84%; *c* 0.22; $CHCl_3$); IR (KBr) *v*: 2981, 1731, 1673, 1601, 1259, 1172, 1115, 841; MS *m*/*z*: 456 (M^+), 411, 364, 321, 275, 229, 135, 107, 77; HRMS: calcd for $C_{25}H_{28}O_8$ $[M + H]^+$ 457.1857, found 457.1863; ^1H NMR (400 MHz, $CDCl_3$) δ 8.06–8.02 (m, 2H), 7.90–7.86 (m, 2H), 6.96–6.92 (m, 2H), 6.92–6.88 (m, 2H), 4.88 (ddd, *J* = 9.10, 6.07, 6.01 Hz, 1H), 4.18–3.99 (m,4H), 3.90 (d, *J* = 9.04 Hz, 1H), 3.85 (s, 3H), 3.84 (s, 3H), 3.51 (dd, *J* = 17.94, 6.02 Hz, 1H), 3.31 (dd, *J* = 17.92, 6.08 Hz, 1H), 1.18 (t, *J* = 7.14 Hz, 3H), 1.15 (t, *J* = 7.14 Hz, 3H); ^{13}C NMR (101 MHz, $CDCl_3$) δ 198.93, 195.07, 168.50, 168.16, 163.72, 163.70, 131.17, 130.42, 129.33, 128.64, 113.88, 113.75, 61.75, 61.67, 55.48, 53.46, 40.19, 38.92, 13.89, 13.85.

Diethyl 2-(1,2-di(*p*-bromobenzoyl)ethyl)propanedioate (3i)

A crude product was purified by column chromatography on silica gel (heptane/EtOAc 10:1) affording a colorless oil. Recrystallization from MeOH led to a white solid (mp 98–100 °C). Enantiomeric excess was determined by HPLC (Chiralpack AS-H column, 9:1 hexane/iPrOH, 1 mL/min, 25 °C, 254 nm. Major isomer t_r = 15.66 min, minor isomer t_r = 19.53 min). $[\alpha]_D^{25}$ = +23.1° (ee = 93%; *c* 0.45; $CHCl_3$); IR (KBr) *v*: 2982, 1731, 1685, 1585, 1398, 1370, 1223, 1178, 1071, 824; MS *m*/*z*: 398, 369, 350, 322, 273, 237, 185, 157, 133, 115, 104, 76; HRMS: calcd for $C_{23}H_{22}Br_2O_6$ $[M + H]^+$ 552.9856, found 552.9867; ^1H NMR (400 MHz, $CDCl_3$) δ 7.87–7.83 (m, 2H), 7.71–7.67 (m, 2H), 7.57–7.53 (m , 2H), 7.53–7.49 (m, 2H), 4.72 (ddd, *J* = 8.78, 7.13, 5.30 Hz, 1H), 4.12–4.04 (m, 2H), 4.04–3.93 (m, 2H), 3.79 (d, *J* = 8.72 Hz, 1H), 3.44 (dd, *J* = 18.22, 5.30 Hz, 1H), 3.31 (dd, *J* = 18.20, 7.08 Hz, 1H), 1.15 (t, *J* = 7.15 Hz, 3H), 1.09 (t, *J* = 7.13 Hz, 3H); ^{13}C NMR (101 MHz, $CDCl_3$) δ 199.57, 195.90, 168.18, 167.94, 134.89, 134.80, 132.18, 132.16, 130.45, 129.77, 128.96, 128.82, 62.11, 62.09, 53.38, 40.45, 39.30, 14.08, 13.99.

Diethyl 2-(1,2-di(*p*-nitrobenzoyl)ethyl)propanedioate (3j)

A crude product was purified by column chromatography on silica gel (heptane/EtOAc 5:1) affording a colorless oil. Recrystallization from iPrOH gave a white solid (mp 94–96 °C). Enantiomeric excess was determined by HPLC (Chiralpack AS-H column, 7:2:1 hexane/iPrOH/EtOH, 0.8 mL/min, 30 °C, 254 nm. Major isomer t_r = 27.54 min, minor isomer t_r = 37.13 min). $[\alpha]_D^{25}$ = +38.9° (ee = 89%; *c* 0.33; $CHCl_3$); IR (KBr) *v*: 2985, 1731, 1690, 1604, 1527, 1347, 1030, 853, 743; MS *m*/*z*: 486 (M), 411, 396, 326, 277, 244, 202, 150, 133, 115, 104, 92, 76; HRMS: calcd for $C_{23}H_{22}N_2O_8$ $[M + H]^+$ 487.1347, found 487.1356; ^1H NMR (400 MHz, $CDCl_3$) δ 8.30–8.26 (m, 2H), 8.25–8.21 (m, 2H), 8.18–8.14 (m, 2H), 8.01–7.97 (m, 2H), 4.73 (ddd, *J* = 8.46, 7.54, 5.27 Hz, 1H), 4.17–4.10 (m, 2H), 4.08–3.95 (m, 2H),

3.81 (d, J = 8.52 Hz, 1H), 3.55 (dd, J = 18.36, 5.28 Hz, 1H), 3.49 (dd, J = 18.30, 7.50 Hz, 1H), 1.20 (t, J = 7.12 Hz, 3H), 1.11 (t, J = 7.12 Hz, 3H); ^{13}C NMR (101 MHz, $CDCl_3$) δ 199.26, 195.55, 167.67, 167.53, 150.67, 150.43, 140.92, 140.16, 129.82, 129.24, 123.98, 123.90, 62.27, 62.27, 53.33, 40.70, 40.11, 14.01, 13.88.

General procedure for the synthesis of racemates 3a-j

A mixture of unsaturated 1,4-diketone **1** (0.25 mmol), malonate **2** (0.37 mmol) and K_2CO_3 (0.25 mmol) in acetone (1 mL) was stirred at 7 °C until TLC showed that all 1,4-diketone was consumed. The product was purified by column chromatography with heptanes/EtOAc as eluent.

Calculations

Initial conformational analysis was carried out by a systematic search method by using the exchange-correlation functional BP86 and the SV basis set (Turbomole 6.3 software [10].) The most favored conformers were determined by using the Boltzmann distribution based on the relative energy of the conformers with a 5 kJ/mol window. The selected conformers were reoptimized with the inclusion of solvent effects (solvent = CCl_4) at the B3PW91/ 6-311G** level of theory by using the Gaussian 03 software (Table) [11]. A scaling factor of 0.96 was applied to the frequencies of the calculated spectra. The calculated spectra were normalized by using dipole strengths.

Table Cartesian coordinates of the lowest-energy conformer of compound **3a**.

C	3.348945	-4.685740	-1.765500
C	2.209834	-3.786777	-2.178812
H	4.299141	-4.146684	-1.780823
H	3.421058	-5.528028	-2.458679
H	3.188783	-5.081112	-0.759861
O	2.159642	-2.686122	-1.240505
H	1.246680	-4.302895	-2.157226
H	2.350988	-3.377859	-3.183123
C	1.160853	-1.816304	-1.396538
O	0.341423	-1.871660	-2.278238
C	1.242678	-0.720213	-0.340573
C	1.411901	-1.333952	1.043357
O	0.696212	-2.191850	1.498371
O	2.439395	-0.784471	1.690804
C	2.655301	-1.238593	3.047144
C	3.865734	-0.514379	3.583117
H	4.058239	-0.832861	4.611086
H	3.705676	0.566062	3.582916
H	4.752159	-0.735057	2.983605

H	2.794950	-2.322736	3.031329
H	1.755170	-1.022394	3.628389
C	0.033590	0.242163	-0.426389
H	2.157360	-0.158480	-0.562006
C	-1.284784	-0.371307	0.027493
C	-2.458157	0.508131	-0.351712
H	-1.288265	-0.516921	1.111261
H	-1.421861	-1.356930	-0.426169
O	-2.283056	1.566519	-0.927963
C	-3.836154	0.046460	-0.005978
C	-4.082092	-1.150245	0.675372
C	-4.916523	0.852202	-0.382995
C	-5.385609	-1.531937	0.973245
C	-6.216382	0.469805	-0.087038
C	-6.453050	-0.724123	0.592513
H	-7.048615	1.101283	-0.384093
H	-7.470729	-1.024315	0.825889
H	-5.567965	-2.461633	1.503986
H	-3.261176	-1.790192	0.980479
H	-4.709869	1.778485	-0.908959
C	0.358440	1.493060	0.394024
H	-0.066801	0.508003	-1.479277
O	0.024502	1.549685	1.562171
C	1.162074	2.590835	-0.222250
C	1.399245	2.693610	-1.597018
C	1.690956	3.565110	0.633690
C	2.154153	3.746550	-2.102395
C	2.448674	4.610992	0.129657
C	2.682622	4.703313	-1.241637
H	1.489809	3.480035	1.696287
H	2.324207	3.822603	-3.172229
H	0.982581	1.969977	-2.288924
H	3.272900	5.524226	-1.638941
H	2.857151	5.358636	0.803305

B3PW91/6-311G**calculated energies of the lowest-energy conformer

Electronic energy: −1342.1080678

Zero Point Energy: 0.4273237

Total Energy: −1341.680743

Gibbs Free Energy: −1341.747248

References

1) Conant, J. B.; Lutz, R. E. *J. Am. Chem. Soc.* **1923**, *45*, 1301-1307.
2) Floyd, M. B.; Du, M. T.; Fabio, P. F.; Jacob, L. A.; Johnson, B. D. *J. Org. Chem.* **1985**, *50*, 5022-5027.

3) Kuang, Y.; Liu, X.; Chang, L.; Wamg, M.; Lin, L.; Feng, X. *Org. Lett.* **2011**, *13*, 3814-3817.
4) Wright, S. W.; Hageman, D. L.; Wright, A. S.; McClure, L. D. *Tetrahedron Lett.* **1997**, *38*, 7345-7348.
5) Jabin, I.; Revial, G.; Monnier-Benoit, N.; Netchitailo, P. *J. Org. Chem.* **2001**, *66*, 256-261.
6) Park, H.-G.; Jeong, B.-S.; Yoo, M.-S.; Lee, J.-H.; Park, M.-K.; Lee, Y.-J.; Kim, M.-J.; Jew, S.-S. *Angew. Chem. Int. Ed.* **2002**, *41*, 3036-3038.
7) Vakulya, B.; Varga, S.; Csampai, A.; Tibor, S. *Org. Lett*. **2005**, *7*, 1967-1969.
8) Malerich, J. P.; Hagihara, K.; Rawal, V. H. *J. Am. Chem. Soc*. **2008**, *130*, 14416-14417.
9) Lee, J. W.; Ryu, T. H.; Oh, J. S.; Bae, H. Y.; Janga, H. B.; Song, C. E. *Chem. Commun*. **2009**, 7224–7226.
10) Ahlrichs, R.; Bär, M.; Häser, M.; Horn, H.; Kölmel, C., *Chem. Phys. Lett*. **1989**, *162*, 165–169.
11) Gaussian 03, Revision E.01, Frisch, M. J.; Trucks, G. W.; Schlegel, H. B.; Scuseria, G. E.; Robb, M. A.; Cheeseman, J. R.; Montgomery, Jr., J. A.; Vreven, T.; Kudin, K. N.; Burant, J. C.; Millam, J. M.; Iyengar, S. S.; Tomasi, J.; Barone, V.; Mennucci, B.; Cossi, M.; Scalmani, G.; Rega, N.; Petersson, G. A.; Nakatsuji, H.; Hada, M.; Ehara, M.; Toyota, K.; Fukuda, R.; Hasegawa, J.; Ishida, M.; Nakajima, T.; Honda, Y.; Kitao, O.; Nakai, H.; Klene, M.; Li, X.; Knox, J. E.; Hratchian, H. P.; Cross, J. B.; Bakken, V.; Adamo, C.; Jaramillo, J.; Gomperts, R.; Stratmann, R. E.; Yazyev, O.; Austin, A. J.; Cammi, R.; Pomelli, C.; Ochterski, J. W.; Ayala, P. Y.; Morokuma, K.; Voth, G. A.; Salvador, P.; Dannenberg, J. J.; Zakrzewski, V. G.; Dapprich, S.; Daniels, A. D.; Strain, M. C.; Farkas, O.; Malick, D. K.; Rabuck, A. D.; Raghavachari, K.; Foresman, J. B.; Ortiz, J. V.; Cui, Q.; Baboul, A. G.; Clifford, S.; Cioslowski, J.; Stefanov, B. B.; Liu, G.; Liashenko, A.; Piskorz, P.; Komaromi, I.; Martin, R. L.; Fox, D. J.; Keith, T.; Al-Laham, M. A.; Peng, C. Y.; Nanayakkara, A.; Challacombe, M.; Gill, P. M. W.; Johnson, B.; Chen, W.; Wong, M. W.; Gonzalez, C.; and Pople, J. A.; Gaussian, Inc., Wallingford CT, 2004.
